# Statistical inference in randomized consent designs in the presence of Hawthorne

**DOI:** 10.1186/1745-6215-16-S2-P137

**Published:** 2015-11-16

**Authors:** Kyungmann Kim

**Affiliations:** 1 University of Wisconsin-Madison, Madison, WI, USA

## 

Randomized consent designs have been advocated in some setting. It has been argued that the use of the randomized consent designs is justified as it eliminates the so-called Hawthorne effect in the control group. Besides the ethical issue in not informing the patients, there are other potentially serious issues regarding the confounding and resultant bias in treatment comparison. This is particularly so in behavioral intervention trials. There is not only the placebo effect, but also the so-called Hawthorn effect that could muddy the comparisons. Based on a randomized consent design with control and intervention which results in three distinct groups, this presentation will show using very simple statistical contrasts why unbiased comparison is impossible due to confounding and self-selection.

